# Regulation and Gene Expression Profiling of NKG2D Positive Human Cytomegalovirus-Primed CD4^+^ T-Cells

**DOI:** 10.1371/journal.pone.0041577

**Published:** 2012-08-01

**Authors:** Helle Jensen, Lasse Folkersen, Søren Skov

**Affiliations:** 1 Section of Biomedicine, Laboratory of Immunology, Faculty of Life Sciences, University of Copenhagen, Copenhagen, Denmark,; 2 Atherosclerosis Research Unit, Department of Medicine, Karolinska Institute, Stockholm, Sweden; Karolinska Institutet, Sweden

## Abstract

NKG2D is a stimulatory receptor expressed by natural killer (NK) cells, CD8^+^ T-cells, and γδ T-cells. NKG2D expression is normally absent from CD4^+^ T-cells, however recently a subset of NKG2D^+^ CD4^+^ T-cells has been found, which is specific for human cytomegalovirus (HCMV). This particular subset of HCMV-specific NKG2D^+^ CD4^+^ T-cells possesses effector-like functions, thus resembling the subsets of NKG2D^+^ CD4^+^ T-cells found in other chronic inflammations. However, the precise mechanism leading to NKG2D expression on HCMV-specific CD4^+^ T-cells is currently not known. In this study we used genome-wide analysis of individual genes and gene set enrichment analysis (GSEA) to investigate the gene expression profile of NKG2D^+^ CD4^+^ T-cells, generated from HCMV-primed CD4^+^ T-cells. We show that the HCMV-primed NKG2D^+^ CD4^+^ T-cells possess a higher differentiated phenotype than the NKG2D^–^ CD4^+^ T-cells, both at the gene expression profile and cytokine profile. The ability to express NKG2D at the cell surface was primarily determined by the activation or differentiation status of the CD4^+^ T-cells and not by the antigen presenting cells. We observed a correlation between CD94 and NKG2D expression in the CD4^+^ T-cells following HCMV stimulation. However, knock-down of CD94 did not affect NKG2D cell surface expression or signaling. In addition, we show that NKG2D is recycled at the cell surface of activated CD4^+^ T-cells, whereas it is produced *de novo* in resting CD4^+^ T-cells. These findings provide novel information about the gene expression profile of HCMV-primed NKG2D^+^ CD4^+^ T-cells, as well as the mechanisms regulating NKG2D cell surface expression.

## Introduction

NKG2D is a type II lectin-like receptor that triggers NK cell activation and co-stimulates γδ T-cells and CD8^+^ T-cells [1], [2]. NKG2D expression is normally absent from CD4^+^ T-cells, however subsets of NKG2D^+^ CD4^+^ T-cells have been found in certain autoimmune diseases [3]–[6]. Recently, a similar subset of NKG2D^+^ CD4^+^ T-cells has been identified, which is specific for HCMV [7]. HCMV is a β-herpesvirus that usually causes lifelong asymptomatic infections in immunocompetent individuals but life-threatening infections in neonates and immunocompromised individuals [8].

The ligands for human NKG2D include MICA, MICB, ULBP1-3, RAET1E, RAET1G, and RAET1L [9], [10]. NKG2D-ligands are either absent or expressed at low levels on healthy cells, but can be induced by cellular stress caused by tumor transformation or infection [10], [11]. The NKG2D/NKG2D-ligand system is frequently used by the immune system to recognize danger signals [10], [11]. However, the system must be kept under strict control to avoid aberrant killing and tissue damage. Anomalous stimulation of the NKG2D/NKG2D-ligand system has been implicated in various autoimmune diseases, including crohn’s disease, celiac disease and rheumatoid arthritis [3], [5], [12]. In contrast, malfunctioning of the system may lead to tumor progression [13], [14]. Thus, modulation of NKG2D expression may hold therapeutic potential for a variety of diseases.

The importance of the NKG2D/NKG2D-ligand system in controlling HCMV infection is apparent through the several mechanisms that HCMV has evolved to evade this system. The HCMV-encoded proteins UL16 and UL142 jointly bind to and sequester NKG2D-ligands in the endoplasmic reticulum (ER)/cis-golgi complex, with exception of the truncated MICA*008 allele, thus preventing cell surface expression of the ligands [9], [15]–[18]. In addition, HCMV encodes a microRNA (i.e. hcmv-miR-UL112), which down-regulates MICB expression by targeting a specific site in the *MICB* 3′ untranslated region [19], [20].

The precise mechanism regulating NKG2D expression on CD4^+^ T-cells is currently undefined and it is possible that several mechanisms can lead to NKG2D expression. Groh V. *et al*. [5] has shown that treatment with IL-15 or TNF-α induces NKG2D cell surface expression on CD4^+^ T-cells from healthy donors and patients with rheumatoid arthritis. Furthermore, Yang D. *et al*. [6] has reported that co-culture with monocytes expressing MIC and membrane-bound IL-15 induces NKG2D cell surface expression on CD4^+^ T-cells from healthy donors. As yet, the gene expression profile of HCMV-specific NKG2D^+^ CD4^+^ T-cells has not been fully studied. In general, NKG2D^+^ CD4^+^ T-cells show no or low expression of the canonical co-stimulatory receptor CD28 [3]–[5], [7]. Additionally, disease-associated NKG2D^+^ CD4^+^ T-cells express IFN-γ, perforin and granzyme B, indicating cytotoxic potential [3]–[5], [7].

CD94 belongs to the C-type lectin superfamily, which also include members of the NKG2 family [21]–[23]. CD94 can form heterodimeric complexes with several proteins of the NKG2 family, generating stimulatory (i.e. CD94-NKG2C or CD94-NKG2E) or inhibitory (i.e. CD94-NKG2A) receptor complexes [23]. CD94 does not pair with NKG2D, which is structurally and functionally unrelated to the other NKG2 family members [23]. CD94/NKG2 receptors are expressed on most NK cells and γδ T-cells, and on subsets of CD8^+^ and CD4^+^ T-cells [23], [24].

In the present study, we used antigen-specific CD4^+^ T-cell-priming in order to study NKG2D cell surface expression on various pathogen-primed CD4^+^ T-cells. Of the pathogens tested, NKG2D cell surface expression was only observed on HCMV-primed CD4^+^ T-cells. HCMV-priming of CD4^+^ T-cells was subsequently used as an approach for studying the regulation and gene expression profile of NKG2D^+^ CD4^+^ T-cells. In this study we provide novel information about the gene expression profile of HCMV-primed NKG2D^+^ CD4^+^ T-cells using genome-wide analysis of individual genes and GSEA. Additionally, we examined the mechanisms regulating NKG2D cell surface expression on CD4^+^ T-cell, as well as the potential role of CD94 in NKG2D cell surface expression and signaling.

## Results

### NKG2D Cell Surface Expression on CD4^+^ T-cells after Co-culture with Autologous Pathogen-pulsed Monocytes

**Figure 1:**
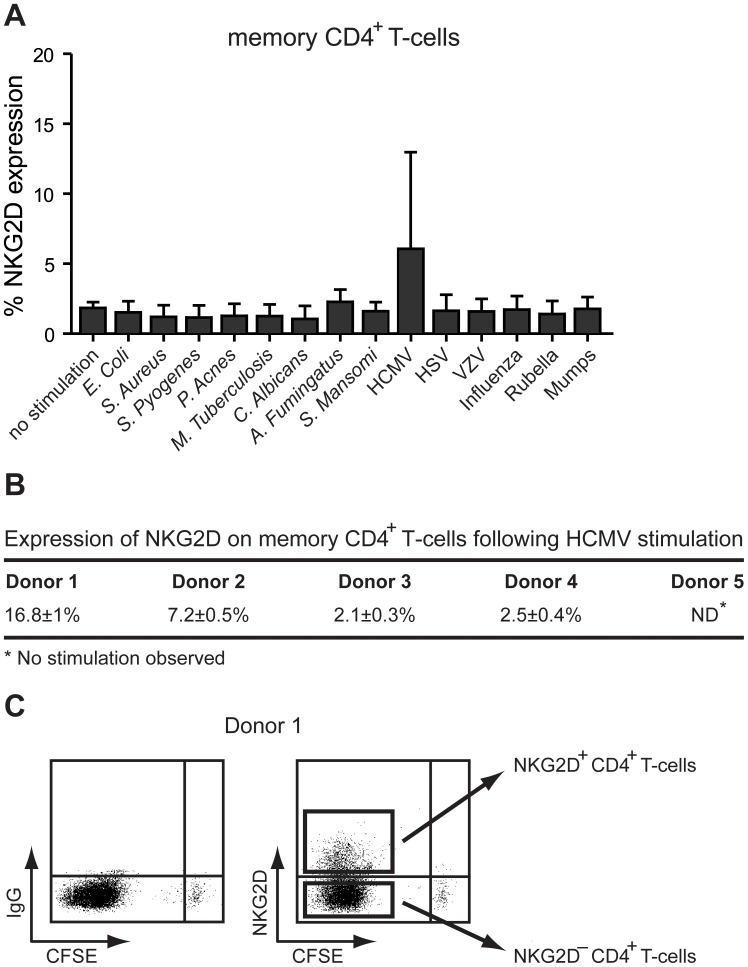
NKG2D cell surface expression on CD4^+^ T-cells after co-culture with autologous pathogen-pulsed monocytes. (**A**), Memory CD4^+^ T-cells were sorted, labeled with CFSE, and stimulated by autologous monocytes pulsed with the indicated inactivated pathogens. 50 U/ml IL-2 was added at day 6 post stimulation. At day 13, stimulated CD4^+^ T-cells (i.e. CFSE negative) were analyzed for cell surface expression of NKG2D by flow cytometry analysis. The bar graphs show mean±SD from five donors. (**B**), Shown is the percentage (mean±SD of two experiments) of NKG2D cell surface expression on CD4^+^ T-cells from the five donors used in (**A**) following priming with HCMV. (**C**), Shown is the scheme for sorting of NKG2D^+^ CD4^+^ T-cells and NKG2D^–^ CD4^+^ T-cells from the CFSE^–^ pool of HCMV-primed memory CD4^+^ T-cells obtained from donor 1. The same approach was used for the cells obtained from donor 2.

A novel approach to obtain antigen-specific T-cell-priming has recently been developed by Zielinski *et al*. [25]. We utilized this approach to study NKG2D cell surface expression on various pathogen-stimulated CD4^+^ T-cells. CD4^+^ T-cells were isolated by magnetic beads from PBMCs obtained from healthy blood donors. Naïve and memory CD4^+^ T-cells, which were sorted according to the expression of CD45RA and CCR7 cell surface markers [26], were labeled with carboxyfluorescein diacetate succinimidyl diester (CFSE) and stimulated for 13 days with autologous monocytes pulsed with various inactivated pathogens (i.e. *Escherichia coli* (*E. coli*); *Staphylococcus aureus* (*S. Aureus*); *Streptococcus pyogenes* (*S. Pyogenes*); *Propionibacterium acnes* (*P. Acnes*); *Mycobacterium tuberculosis* (*M. Tuberculosis*); *Candida albicans* (*C. Albicans*); *Aspergillus Fumingatus* (*A. Fumingatus*); *Schitsomi mansomi* (*S. Mansomi*); HCMV; Herpes simplex virus I+II (HSV); Varicella-zoster virus (VZV); Influenza; Rubella virus; and Mumps virus). As shown in Figure 1A, NKG2D cell surface expression was solely observed on HCMV-primed memory CD4^+^ T-cells. Four out of the five donors tested showed proliferation of memory CD4^+^ T-cells following priming with HCMV, suggesting a previous infection (data not shown). However, only two (i.e. donor 1 and 2) of these four donors displayed NKG2D^+^ CD4^+^ T-cells (Fig. 1B). Donor 1 displayed NKG2D^+^ CD4^+^ T-cells following priming of both naïve (data not shown) and memory CD4^+^ T-cells with HCMV (Fig. 1B), whereas donor 2 displayed NKG2D^+^ CD4^+^ T-cells only following HCMV-priming of memory CD4^+^ T-cells (data not shown and Fig. 1B). Interestingly, priming with HSV or VZV, which belong to the same herpesvirus family as HCMV, did not induce NKG2D cell surface expression on naïve (data not shown) or memory CD4^+^ T-cells (Fig. 1A).

Together these results suggest that the induction of NKG2D cell surface expression on CD4^+^ T cells following HCMV stimulation is an explicit event, involving a stimulus specific for HCMV.

### Genome-wide Analysis of Individual Genes of HCMV-primed NKG2D^+^ CD4^+^ T-cells

To investigate the gene expression profile of CD4^+^ T-cells expressing NKG2D, we sorted NKG2D^+^ CD4^+^ T-cells and NKG2D^–^ CD4^+^ T-cells from the CFSE^–^ pool of HCMV-primed memory CD4^+^ T-cells obtained from donor 1 (Fig. 1C) and donor 2 (data not shown). The total RNA was isolated and used for microarray analysis. From the microarray analysis we identified 300 genes with an average of at least 1.5-fold change (127 up-regulated and 173 down-regulated) in the NKG2D^+^ CD4^+^ T-cells relative to the NKG2D^−^ CD4^+^ T-cells from the two donors (Table S1). As expected, NKG2D was highly expressed in the sorted NKG2D^+^ CD4^+^ T-cells, but absent in the NKG2D^–^ CD4^+^ T-cells (Table 1 and Table S1). From the microarray analysis, we found that the two donors displayed a different expression pattern of certain known stimulatory receptors (e.g. 2B4, NKp46/30/80, and CD161) and inhibitory receptors (e.g. ILT-2, killer-cell immunoglobulin-like receptors (CD158a-j), and CTLA4) (Table 1). However, expression of the co-stimulatory receptors CD28 and CD27 was highly decreased in the NKG2D^+^ CD4^+^ T-cells from both donors (Table 1).

**Table 1:** Expression of genes encoding immune regulatory receptors.

		Expression (fold)	
Gene	Gene product	Donor 1	Donor 2
*CD244*	Natural killer cell receptor 2B4	1.82	3.04
*CD27*	CD27 (co-stimulatory receptor)	−2.63	−4.15
*CD28*	CD28 (co-stimulatory receptor)	−2.70	−3.84
*KLRB1*	Killer cell lectin-like receptor (CD161)	−1.62	−3.59
*KLRC2*	Killer cell lectin-like receptor (NKG2C)	1.21*	1.13*
*KLRC3*	Killer cell lectin-like receptor (NKG2E)	2.88*	1.19*
*KLRC4*	Killer cell lectin-like receptor (NKG2F)	3.39*	−1.03*
*KLRD1*	Killer cell lectin-like receptor (CD94)	6.06	12.08
*KLRF1*	Killer cell lectin-like receptor (NKp80)	2.17	1.61
*KLRK1*	Killer cell lectin-like receptor (NKG2D)	10.92	20.95
*NCR1*	Natural cytotoxicity triggering receptor 1 (NKp46)	1.18	1.97
*NCR3*	Natural cytotoxicity triggering receptor 3 (NKp30)	2.73	1.41
*CTLA4*	Cytotoxic T-lymphocyte-associated protein 4 (CTLA-4)	−4.68	−1.25
*KIR2DL1*	Killer cell immunoglobulin-like receptor (CD158a)	1.87	2.33
*KIR2DL2*	Killer cell immunoglobulin-like receptor (CD158b1)	1.95	1.97
*KIR2DL3*	Killer cell immunoglobulin-like receptor (CD158b2)	4.19	2.29
*KIR2DL4*	Killer cell immunoglobulin-like receptor (CD158d)	1.82	1.53
*KIR2DS1*	Killer cell immunoglobulin-like receptor (CD158h)	2.29	1.17
*KIR2DS2*	Killer cell immunoglobulin-like receptor (CD158j)	3.20	1.13
*KIR3DL1*	Killer cell immunoglobulin-like receptor (CD158e1)	3.15	1.40
*KIR3DS1*	Killer cell immunoglobulin-like receptor (CD158e2)	4.84	1.08
*KIR3DX1*	Killer cell immunoglobulin-like receptor	1.74	2.66
*KLRC1*	Killer cell lectin-like receptor (NKG2A)	1.63*	−1.24*
*LILRB1*	Leukocyte immunoglobulin-like receptor (ILT-2)	1.80	1.73

The values represent fold change of gene expression obtained from the microarray analysis, when comparing the NKG2D^+^ CD4^+^ T-cells with the NKG2D^−^ CD4^+^ T-cells of donor 1 and 2. Positive values correspond to genes up-regulated in the NKG2D^+^ CD4^+^ T-cells compared to NKG2D^−^ CD4^+^ T-cells, whereas negative values correspond to genes down-regulated.

* denote genes with low expression values (<35 units) in both CD4+ T-cell populations.

Interestingly, expression of CD94 was potently up-regulated in the NKG2D^+^ CD4^+^ T-cells from both donors compared to the NKG2D^–^ CD4^+^ T-cells (Table 1 and Table S1), whereas the expression value of the known binding partners of CD94 (i.e. NKG2A/C/E/F) [23] was low (<35 units) in both CD4^+^ T-cell populations (Table 1).

The NKG2D expression was correlated with a higher expression level of certain effector molecules (i.e. granulysin [27], perforin [28] and FGFBP2 [29]) in both donors (Table S1). Furthermore, expression of CD25 (interleukin-2 receptor α (IL-2Rα)) and CD127 (IL-7R) was down-regulated in the NKG2D^+^ CD4^+^ T-cells from both donors compared to the NKG2D^–^ CD4^+^ T-cells (Table S1), which is indicative of an effector phenotype [30]–[32].

### Gene Set Enrichment Analysis of HCMV-primed NKG2D^+^ CD4^+^ T-cells

**Figure 2:**
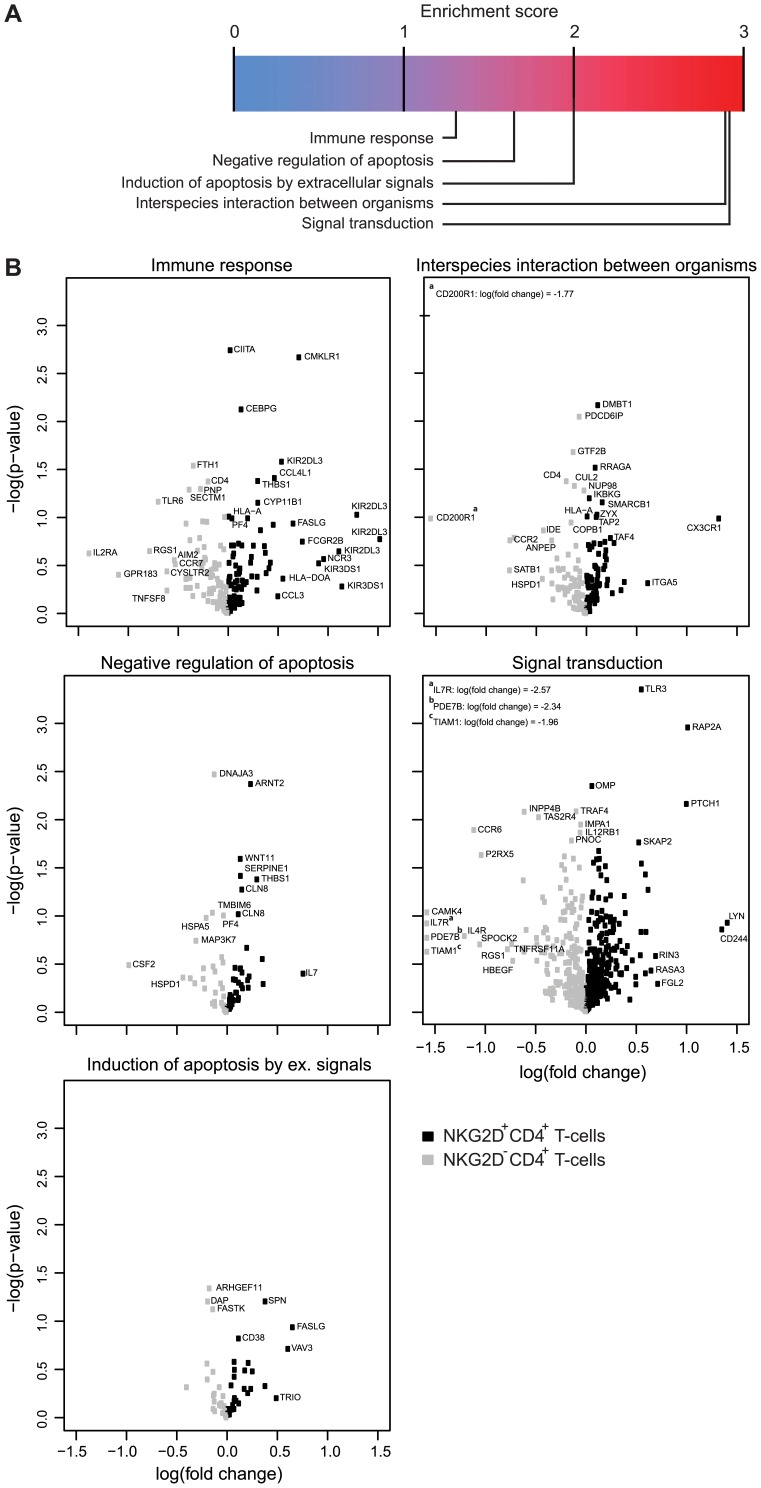
Gene set enrichment analysis of HCMV-primed NKG2D^+^ CD4^+^ T-cells. (**A**) Gene set enrichment analysis was performed as described in *Materials and Methods*. The indicated score is the –log10(*P*) of the multiple testing corrected significance. (**B**) Volcano plots are shown for 5 of the 19 gene sets and pathways which were significantly different when comparing NKG2D^+^ CD4^+^ T-cells with NKG2D^–^ CD4^+^ T-cells. Volcano plots of significant gene sets were created by plotting the significance of expression change using a paired Student’s *t*-test and the fold-change as the log of ratio between the average expression values in each group. Black squares indicate genes that have higher expression in the NKG2D^+^ CD4^+^ T-cells, whereas gray squares indicate genes that have higher expression in the NKG2D^–^ CD4^+^ T-cells.

To further analyze the gene expression profile of the NKG2D^+^ CD4^+^ T-cells, we performed a GSEA. GSEA is a pathway-based analysis that detects changes in expression of genes in entire pathways and is far more robust than analyses based upon individual genes. GSEA identified 19 gene sets and pathways that were significantly different when comparing the microarray data of the NKG2D^+^ CD4^+^ T-cells and NKG2D^–^ CD4^+^ T-cells obtained from donor 1 and 2 (data not shown). We chose to look further into five of these pathways: *immune response*, *negative regulation of apoptosis*, *induction of apoptosis by extracellular signals*, *interspecies interaction between organisms*, and *signal transduction* (Fig. 2A), which could relate to the immune function of the NKG2D^+^ CD4^+^ T-cells.

We observed that the NKG2D^+^ CD4^+^ T-cells expressed higher levels of the effector molecule FASLG [33] (*immune response* & *induction of apoptosis by extracellular signals*, Fig. 2B) and the chemokine receptor CX3CR1 (*interspecies interaction between organisms*, Fig. 2B), expression of which is associated with effector CD4^+^ T-cells [34], [35]. In addition, the two cell surface markers CCR7 and CD62L (L-selectin), loss of which define an effector memory T-cell subset [36], were expressed at a lower level in the NKG2D^+^ CD4^+^ T-cells compared to the NKG2D^–^ CD4^+^ T-cells (*immune response*, Fig. 2B and Table S1).

Overall these data indicate that the HCMV-primed NKG2D^+^ CD4^+^ T-cells appear more differentiated and “effector-like” than the NKG2D^–^ CD4^+^ T-cells.

### Involvement of CD94 in NKG2D Signaling and Cell Surface Expression on CD4^+^ T-cells

From the microarray analysis, we found that CD94 was highly expressed in the HCMV-primed NKG2D^+^ CD4^+^ T-cells, and this expression did not correlate with the expression level of the known binding partners of CD94 (i.e. NKG2A/C/E/F) [23] (Table 1). We therefore speculated whether CD94 plays a role in NKG2D cell surface expression and/or signaling.

**Figure 3:**
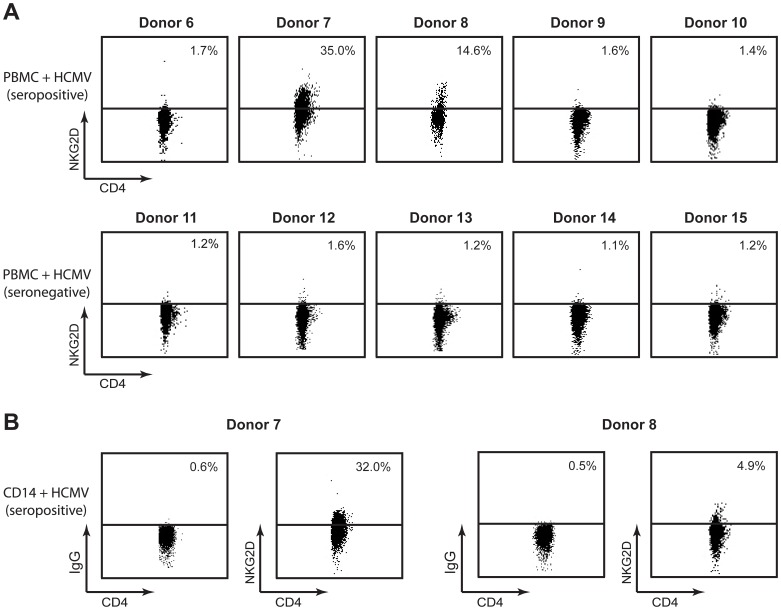
NKG2D cell surface expression on CD4^+^ T-cells correlates with the HCMV serostatus of the donors. (**A**) PBMCs, isolated from five HCMV seropositive and five HCMV seronegative donors, were stimulated with HCMV. NKG2D cell surface expression of CD4^+^ T-cells was examined by flow cytometry at day 13 post stimulation. (**B**) CD4^+^ T-cells were stimulated by autologous monocytes pulsed with HCMV. NKG2D cell surface expression on CD4^+^ T-cells was examined by flow cytometry at day 13 post stimulation. The dot plots represent one out of three experiments.

**Figure 4:**
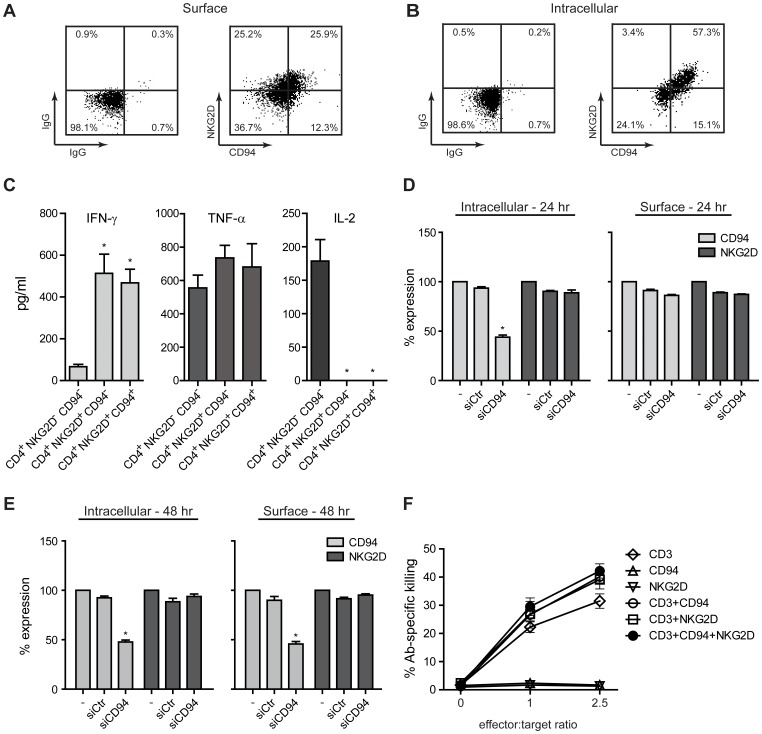
Involvement of CD94 in NKG2D signaling and cell surface expression on HCMV-primed CD4^+^ T-cells. (**A,B**) PBMCs (donor 7) were stimulated with HCMV for 13 days. CD4^+^ T-cells were analyzed for cell surface and intracellular expression of NKG2D and CD94 by flow cytometry. The dot plots represent one out of three experiments. The bar graphs show mean±SD from three experiments. (**C**) NKG2D^–^ CD94^–^, NKG2D^+^ CD94^–^ and NKG2D^+^ CD94^+^ CD4^+^ T-cells were sorted from PBMCs (donor 7) stimulated with HCMV for 13 days. The cells were incubated with ionomycin/PMA for 5 hr and the supernatants were analyzed for cytokine expression by multiplexed cytokine analysis. The bar graphs show mean±SD from duplicate measurements of the same samples and are representative of one out of three experiments. (**D+E**) CD4^+^ T-cells (donor 7), stimulated with autologous monocytes pulsed with HCMV, were either non-transfected (−) or transfected with control siRNA (siCtr) or a siRNA mixture directed against CD94 (siCD94). Cell surface and intracellular expression of CD94 and NKG2D were assessed by flow cytometry 24 and 48 hr post transfection. The percentage of NKG2D and CD94 expression is given as a comparison between the non-transfected sample and the siCtr or siCD94 samples, where the expression in the non-transfected sample was set to 100%. The bar graphs show mean±SD from two experiments. (**F**) A redirected cytotoxicity assay was performed as described in *Materials and Methods*. Shown is the mean±SD from two experiments. *indicates p<0.05.

Peripheral blood mononuclear cells (PBMCs) from five HCMV seropositive donors and five HCMV seronegative donors were stimulated with HCMV and the CD4^+^ T-cells were analyzed for NKG2D and CD94 expression. As shown in Fig. 3A, NKG2D cell surface expression was observed in only two out of the ten donors tested (i.e. donor 7 and 8), both of which were HCMV seropositive. We saw the same results when stimulating the PBMCs with inactivated lysate of HCMV infected cells (cmv 2) or inactivated and purified HCMV (cmv β) (data not shown) or when stimulating CD4^+^ T-cells isolated from the ten donors with autologous monocytes pulsed with inactivated HCMV (Fig. 3B and data not shown). Similarly, CD94 expression was solely detected on HCMV-primed CD4^+^ T-cells obtained from donor 7 and 8 (Fig. 4A, C, B and data not shown, respectively). A correlation between the cell surface and intracellular expression of CD94 and NKG2D was observed in the HCMV-primed CD4^+^ T-cells obtained from donor 7 (Fig. 4A, C, B) and donor 8 (data not shown). No cell surface expression of NKG2A or NKG2C was detected on the HCMV-primed CD4^+^ T-cells from both donors (data not shown), suggesting that CD94 has another binding partner or is expressed at the cell surface as a homodimeric receptor complex.

To assess whether the cell surface expression of CD94 defines a distinct population of the HCMV-primed NKG2D^+^ CD4^+^ T-cells, we sorted NKG2D^–^ CD94^–^, NKG2D^+^ CD94^–^, and NKG2D^+^ CD94^+^ CD4^+^ T-cells from PBMCs stimulated with HCMV. The sorted cells were incubated with ionomycin/phorbol 12-myristate 13-acetate (PMA) for 5 hr and the supernatants were analyzed for cytokine expression. No difference was detected in the cytokine profile of NKG2D^+^ CD94^–^ CD4^+^ T-cells versus NKG2D^+^ CD94^+^ CD4^+^ T-cells (Fig. 4C). However, we observed a notable difference in the cytokine profile of the NKG2D^+^ CD4^+^ T-cells versus NKG2D^–^ CD4^+^ T-cells (Fig. 4C). The cytokine profile of the NKG2D^+^ CD4^+^ T-cells resembled that of highly differentiated CD4^+^ T-cells (i.e. high IFN-γ and no/low IL-2 production) [37], whereas the cytokine profile of the NKG2D^–^ CD4^+^ T-cells resembled that of less differentiated CD4^+^ T-cells (i.e. low IFN-γ and high IL-2 production) [37] (Fig. 4C). Expression of TNF-α was slightly increased with the NKG2D^+^ CD4^+^ T-cells compared to the NKG2D^–^ CD4^+^ T-cells (Fig. 4C). Thus, the cytokine profile of the HCMV-primed NKG2D^+^ CD4^+^ T-cells corresponded with the gene expression profile of the cells obtained from the microarray analysis and GSEA.

To examine the importance of CD94 for NKG2D cell surface expression, we transiently transfected HCMV-primed CD4^+^ T-cells derived from donor 7 with control siRNA (siCtr) or a mixture of three different siRNAs directed against CD94 (siCD94). 24 hr post transfection, the intracellular protein level of CD94 was significantly decreased in the siCD94 transfected sample compared to the non-transfected and siCtr sample (Fig. 4D). However, no significant decrease was observed in the cell surface expression of CD94 (Fig. 4D). The intracellular and cell surface level of NKG2D remained unaffected 24 hr post transfection (Fig. 4D). 48 hr post transfection, a significant decrease of both the intracellular and cell surface expression of CD94 was observed (Fig. 4E). However, the intracellular and cell surface level of NKG2D was still not affected (Fig. 4E).

**Figure 5:**
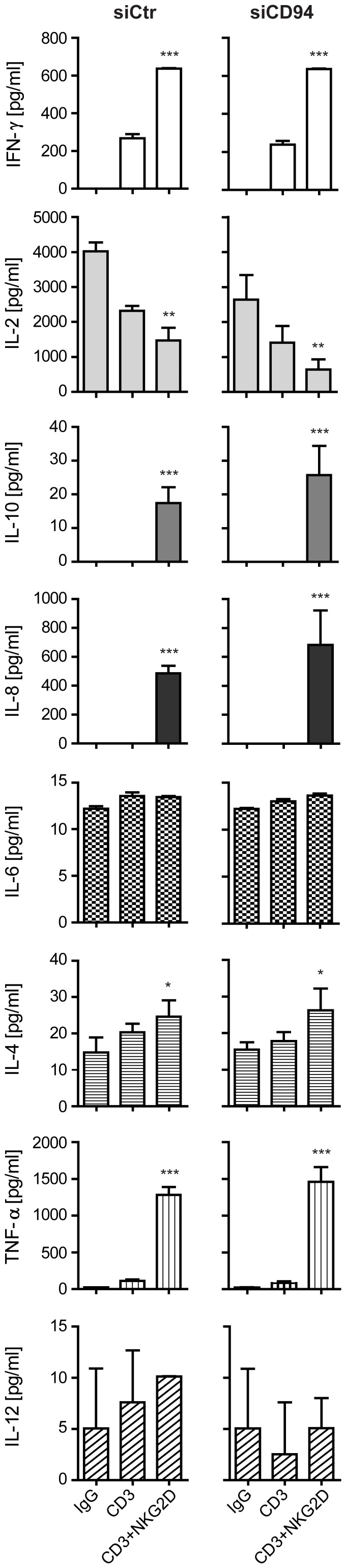
CD94 is not crucial for NKG2D-mediated signaling in HCMV-primed CD4^+^ T-cells. CD4^+^ T-cells transfected with siCtr or siCD94 were stimulated by plate-bound IgG, CD3 or CD3+NKG2D Abs for 96 hr. The supernatants were analyzed for cytokine expression by multiplexed cytokine analysis. The bar graphs show mean±SD from duplicate measurements of two experiments. *indicates p<0.05, **indicates p = 0.0046, and ***indicates p<0.0001.

To assess whether the down-modulation of CD94 affected NKG2D signaling, we stimulated the siRNA transfected cells with plate-bound IgG, anti-CD3 Ab, or anti-CD3 Ab plus anti-NKG2D Ab for 96 hr and analyzed the supernatants for cytokine expression. We did not detect any difference in the cytokine profile between the siCtr or siCD94 transfected CD4^+^ T-cells (Fig. 5). However, we did observe a notable difference in the cytokines expressed following anti-CD3 Ab stimulation alone and anti-CD3 Ab plus anti-NKG2D Ab stimulation (Fig. 5). Stimulation through NKG2D significantly potentiated the expression of IFN-γ and IL-4, whereas the expression of IL-2 was significantly decreased (Fig. 5). In addition, only stimulation with anti-CD3 Ab in combination with anti-NKG2D Ab led to expression of IL-10, IL-8, and TNF-α (Fig. 5). No difference in IL-6 and IL-12 production was observed following the different Ab stimuli (Fig. 5).

Together, these results suggest that the CD94 expression does not influence the cell surface expression of NKG2D or its signaling capacity.

To examine the functional significance of the CD94 expression, we performed a redirected cytotoxicity assay using HCMV-primed CD4^+^ T-cells derived from donor 7 as effector cells and DIO-stained monocytes incubated with different combinations of Abs directed against CD3, CD94 and NKG2D as target cells. Ab-specific killing of the DIO-stained target cells was measured by propidium iodide (PI) uptake after 5 hr and 24 hr. A low level of Ab-specific killing following CD3, CD3+CD94, CD3+NKG2D, and CD3+CD94+NKG2D triggering was observed after 5 hr (data not shown). After 24 hr, the level of the Ab-specific killing was higher (Fig. 4F). As shown in Fig. 4F, triggering of CD3 alone was sufficient to induce cytotoxicity by the HCMV-primed CD4^+^ T-cells. Triggering of CD3 plus CD94 and/or NKG2D resulted in an increased cytotoxicity by the HCMV-primed CD4^+^ T-cells (Fig. 4F). No cytotoxicity was observed following triggering of CD94 or NKG2D alone (Fig. 4F). These results indicate that signaling through NKG2D or CD94 can augment CD3-induced cytotoxicity by the HCMV-primed CD4^+^ T-cells.

### The Ability to Express NKG2D is Determined by the Activation or Differentiation Status of the HCMV-primed CD4^+^ T-cells

**Figure 6:**
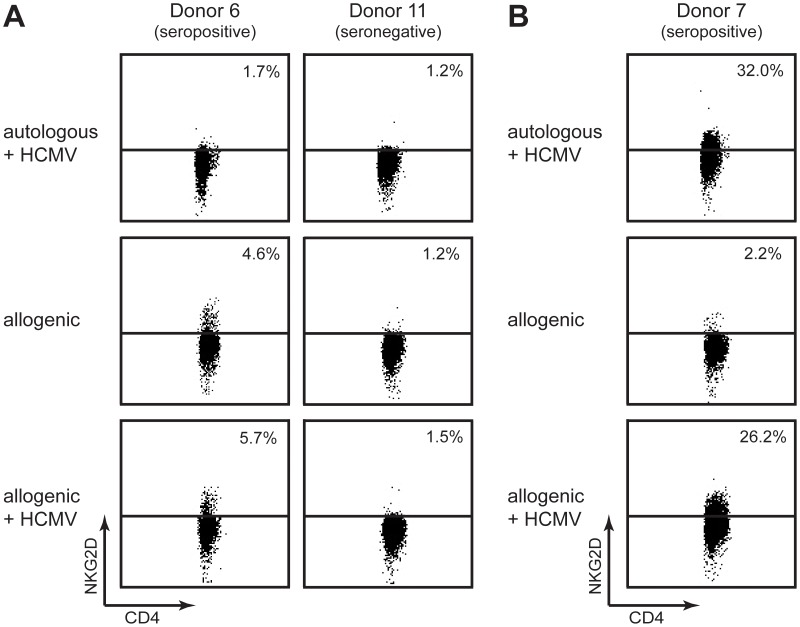
The ability to express NKG2D is determined by the activation or differentiation status of the HCMV-primed CD4^+^ T-cells. CD4^+^ T-cells were stimulated with autologous or allogeneic monocytes +/− pulsed with HCMV. At day 13 post stimulation, NKG2D cell surface expression was analyzed on the CD4^+^ T-cells by flow cytometry. The dot plots represent one out of two experiments. (**A**) Shown are the results from autologous or allogeneic stimulation of CD4^+^ T-cells obtained from donor 6 and 11. The dot plots shown for the donor 11-derived CD4^+^ T-cells (left panel) is representative of the results for the CD4^+^ T-cells obtained from the remaining four donors (i.e. donor 9, 10, 12 and 13). (**B**) Shown are the results from autologous and allogeneic stimulation of CD4^+^ T-cells obtained from donor 7. The dot plots are shown for the allogeneic stimulation with donor 6-derived monocytes. Similar results were obtained when using donor 11-derived monocytes for the allogeneic stimulation.

Monocytes displaying MIC cell surface expression and membrane-bound IL-15 have been suggested to be important for the induction of NKG2D expression on CD4^+^ T-cells [6]. To examine the involvement of monocytes in the induction of NKG2D cell surface expression on CD4^+^ T-cells following HCMV-priming, we isolated CD4^+^ T-cells from three HCMV seropositive donors (i.e. donor 6, 9, and 10 in Fig. 3A) and three HCMV seronegative donors (i.e. donor 11, 12, and 13 in Fig. 3A). The CD4^+^ T-cells were then stimulated with allogeneic monocytes for 13 days with or without HCMV pulse. The allogeneic monocytes used for stimulation was isolated from donor 7, which was the donor displaying the highest level of NKG2D^+^ CD4^+^ T-cells (Fig. 3A). If it is the monocytes that provide the sufficient stimuli for inducing NKG2D expression on CD4^+^ T-cells following HCMV-priming, we would expect to observe a population of NKG2D^+^ CD4^+^ T-cells in this experimental setup. As shown in Figure 6A, we observed a minor population of NKG2D^+^ CD4^+^ T-cells with the HCMV seropositive donor 6, regardless of HCMV pulse. However, we did not detect a population of NKG2D^+^ CD4^+^ T-cells with the remaining five donors (Fig. 6A and data not shown), despite a successful allogeneic stimulation (data not shown). These results suggest that the ability of HCMV-stimulated CD4^+^ T-cells to express NKG2D is not crucial dependent on a specific stimuli provided by the monocytes.

It is possible that the ability to express NKG2D is a consequence of the activation or differentiation status of the CD4^+^ T-cells. To test this hypothesis we used the inverse approach of the experiment, where CD4^+^ T-cells isolated from donor 7 were stimulated with allogeneic monocytes isolated from the HCMV seropositive donor 6 or the HCMV seronegative donor 11, with or without HCMV pulse. As shown in Figure 6B, we observed NKG2D cell surface expression on the CD4^+^ T-cells following allogeneic stimulation with HCMV pulse, but not following allogeneic stimulation alone, which indicates that NKG2D is merely expressed on HCMV-primed CD4^+^ T-cells. NKG2D cell surface expression was induced on the CD4^+^ T-cells regardless of the HCMV serostatus of the donors from which the monocytes were derived (Fig. 6B and data not shown). Furthermore, the level of NKG2D cell surface expression on the allogeneic-stimulated CD4^+^ T-cells resembled that of the stimulation with autologous monocytes (Fig. 6B and data not shown). Importantly, we did not obtain NKG2D cell surface expression when stimulating CD4^+^ T-cells isolated from the HCMV seropositive donors 9 or 10 with the same allogeneic monocytes as above, with or without HCMV pulse (data not shown).

Together these results suggest that the ability to express NKG2D on the cell surface is primarily dependent on the activation or differentiation status of the CD4^+^ T-cells and not the antigen presenting cells. Thus, it appears that infection with HCMV can lead to a change in a population of the CD4^+^ T-cells, making them capable of expressing NKG2D.

### The Dynamics of NKG2D Cell Surface Expression on HCMV-primed CD4^+^ T-cells

**Figure 7:**
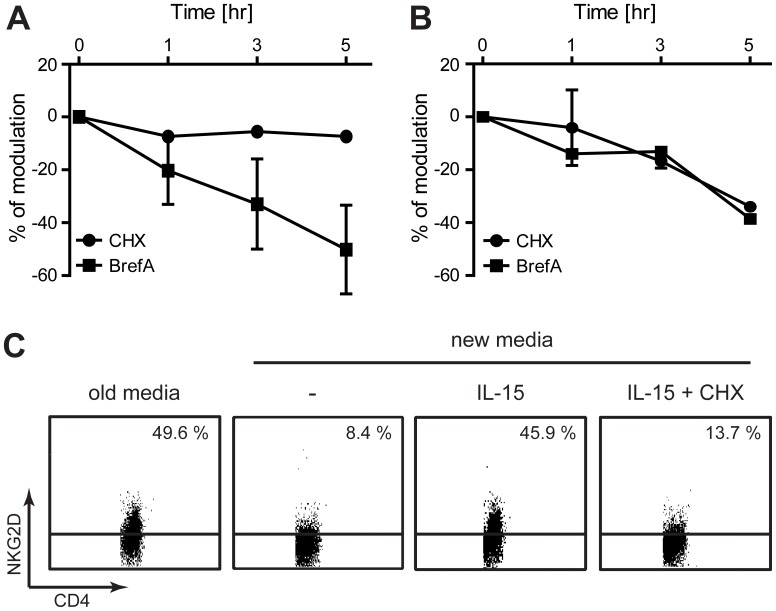
The dynamics of NKG2D cell surface expression on HCMV-primed CD4^+^ T-cells. CD4^+^ T cells were stimulated by autologous monocytes pulsed with HCMV for 11 days (**A**) or 23–25 days (**B**). The cells were treated with CHX (50 µg/ml) or BrefA (10 µg/ml) and NKG2D surface expression was assessed by flow cytometry 0, 1, 3 and 5 hr post treatment. % of modulation was calculated as (% expression of treated cells - % expression of un-treated cells)/% expression of un-treated cells * 100. The graphs show mean±SD from three experiments. (**C**) CD4^+^ T cells were stimulated by autologous monocytes pulsed with HCMV for 23–25 days. Some of the cells were re-cultured in new complete media without cytokines for 24 hr. The “new media” cells were split and either left untreated or treated with 50 ng/ml IL-15+/−0.5 µg/ml CHX for 24 hr. NKG2D cell surface expression on CD4^+^ T-cells was measured by flow cytometry. The dot plots represent one out of three experiments.

The dynamics of NKG2D expression at the cell surface of HCMV-primed CD4^+^ T-cells was studied by treatment with brefeldin A (BrefA) and cycloheximide (CHX). Treatment with BrefA blocks anterograde transport of proteins from the ER to the Golgi complex, as well as protein transport within the secretory/endocytic pathway beyond the Golgi complex [38]. CHX is a potent inhibitor of protein synthesis, thus treatment with BrefA in parallel with CHX permits discrimination between the effects of BrefA on the endocytic pathway and the ER-to-Golgi transport. As shown in Figure 7A, treatment with BrefA, but not CHX, inhibited the NKG2D cell surface expression on activated CD4^+^ T-cells (i.e. day 11 post HCMV-priming). In contrast, both BrefA and CHX treatment decreased the NKG2D cell surface expression on resting CD4^+^ T-cells (i.e. day 23–25 post HCMV-priming) (Fig. 7B). BrefA and CHX worked as expected, as we observed a potent reduction in the cell surface expression of the transferrin receptor CD71 following BrefA treatment, but not following CHX treatment [39] (data not shown). These results suggest that NKG2D is recycled at the cell surface during activation, whereas it is expressed *de novo* in resting CD4^+^ T-cells.

To examine whether the activated and resting CD4^+^ T-cells need an extracellular stimulus to maintain the NKG2D cell surface expression, we replaced the culture media of the cells with new media containing no cytokines. Replacement of media in the sample with activated CD4^+^ T-cells resulted in a small increase in NKG2D cell surface expression (data not shown). In contrast, replacement of media in the sample with resting CD4^+^ T-cells led to a robust decrease in NKG2D cell surface expression (Fig. 7C). The inhibition of NKG2D cell surface expression was partly blocked by treatment with either hypertonic sucrose or methyl-β-cyclodextrin (data not shown), suggesting that NKG2D was down-modulated by clathrin-mediated endocytosis. The loss of NKG2D cell surface expression could be rescued by treatment with IL-15 (Fig. 7C) or IL-2 (data not shown). Furthermore, the IL-15 or IL-2 induced expression of NKG2D could be blocked by treatment with CHX (Fig. 7C and data not shown, respectively). These results indicate that resting, but not the activated, NKG2D^+^ CD4^+^ T-cells need a stimulatory signal, provided by IL-2 or IL-15, in order to sustain the NKG2D cell surface expression.

## Discussion

NKG2D expression is normally absent from CD4^+^ T-cells, however expression has been detected on certain virus-specific CD4^+^ T-cells [7], [40]. In this study we analyzed the cell surface expression of NKG2D on a panel of pathogen-stimulated CD4^+^ T-cells. CFSE-labeled naïve and memory CD4^+^ T-cells were stimulated by autologous monocytes pulsed with various inactivated pathogens, including five bacteria, two fungi, six viruses and one trematode. Of the 14 pathogens tested, NKG2D cell surface expression was solely observed on HCMV-primed CD4^+^ T-cells. VZV- or HSV-primed CD4^+^ T-cells did not display NKG2D cell surface expression, at least not in our experimental setup, despite belonging to the same virus family as HCMV. These results indicate that the induction of NKG2D cell surface expression on CD4^+^ T-cells is a restricted event and not a general consequence of immune activation.

The induction of NKG2D cell surface expression on HCMV-primed CD4^+^ T-cells was influenced by the HCMV serostatus of the donors, as NKG2D^+^ CD4^+^ T-cells were solely obtained from blood donors who had previously encountered HCMV infection. A total of nine HCMV seropositive donors were used in this study, but surprisingly only four of the donors exhibited a subset of NKG2D^+^ CD4^+^ T-cells following priming with HCMV. Monocytes displaying MIC cell surface expression and membrane-bound IL-15 have previously been shown to induce NKG2D cell surface expression on CD4^+^ T-cells [6]. However, the induction of NKG2D cell surface expression on HCMV-primed CD4^+^ T-cells was not dependent on the phenotype of the monocytes, but rather dependent on the activation or differentiation status of the CD4^+^ T-cells. We did not discover the specific change in the CD4^+^ T-cells leading to NKG2D cell surface expression. However, the HCMV-primed NKG2D^+^ CD4^+^ T-cells showed a higher differentiated phenotype than the NKG2D^–^ CD4^+^ T-cells, both at the gene expression profile and cytokine profile, which again indicate that the differentiation status of the CD4^+^ T-cells is an important factor. Highly differentiated CD4^+^ T-cells is a consequence of repeated antigen exposure [37], thus it is possible that the level of HCMV re-infections affect the ability of the CD4^+^ T-cells to express NKG2D. This could also explain the difference in the appearance of NKG2D^+^ CD4^+^ T-cells between the HCMV seropositive donors used in this study.

The majority of NKG2D^+^ CD4^+^ T-cell subsets identified so far express no or low levels of CD28, which is correlated with a highly differentiated phenotype [3]–[5], [7]. These CD28^–^ NKG2D^+^ CD4^+^ T-cell are IFN-γ and TNF-α producing cytotoxic T-cells [3]–[5], [7]. However, a distinct subset of NKG2D^+^ CD4^+^ T-cells have been identified, which are immunosuppressive and lack proinflammatory cytokine and cytotoxic signatures [41]. Our results indicate the HCMV-primed NKG2D^+^ CD4^+^ T-cells to be highly differentiated. Thus, it was surprising that we observed NKG2D cell surface expression after priming of naïve CD4^+^ T-cells obtained from donor 1 (but not donor 2) with HCMV. It is possible that distinct NKG2D^+^ CD4^+^ T-cell populations can be generated following HCMV priming.

The precise mechanism of induction of NKG2D expression on HCMV-primed CD4^+^ T-cells is currently not known. Expression of thymocyte selection-associated high mobility group box (TOX), which is a transcription factor required for the differentiation of CD4^+^ T-cell and NK cell lineages [42], [43], was found to be up-regulated in the HCMV-primed NKG2D^+^ CD4^+^ T-cells compared to the NKG2D^–^ CD4^+^ T-cells in the microarray analysis (Table S1). Thus, it would be interesting to examine the involvement of TOX in the differentiation of CD4^+^ T-cells capable of expressing NKG2D.

We found a correlation between CD94 and NKG2D expression in the HCMV-primed CD4^+^ T-cells. Nonetheless, we found no indication that CD94 expression was important for NKG2D surface expression and/or signaling in these cells. CD94 can form heterodimeric receptor complexes through covalent binding of proteins belonging to the NKG2 family, except NKG2D [23]. However, we were not able to detect surface expression of NKG2A or NKG2C on the HCMV-primed CD4^+^ T-cells, suggesting that CD94 has another binding partner or is expressed at the cell surface as a homodimeric receptor complex.

Expression of human CD94 is controlled by dual promoters, which show differential sensitivity towards IL-2 and IL-15 [44]. Similarly, human *NKG2D* possesses multiple transcriptional start sites [22], which suggest the possible use of dual promoters. Our results indicate that CD94 and NKG2D expression might be regulated similarly in CD4^+^ T-cells following priming with HCMV. Thus, delineating the mechanism of induction of CD94 expression could reveal important information about the regulation of NKG2D expression in the HCMV-primed CD4^+^ T-cells and vice versa. It would be interesting to examine if the expression of CD94 and NKG2D is correlated in other cell types, such as NK cells and CD8^+^ T-cells, as well as other subsets of disease-associated NKG2D^+^ CD4^+^ T-cells.

When studying the dynamics of NKG2D cell surface expression on the HCMV-primed CD4^+^ T-cells, we found that NKG2D was recycled on the cell surface of the CD4^+^ T-cells during activation. In contrast, NKG2D was produced *de novo* in resting CD4^+^ T-cells, and a stimulatory signal provided by either IL-2 or IL-15 was needed in order to sustain the cell surface expression of NKG2D. These results indicate that cell surface expression of NKG2D is important for the effector function of the activated CD4^+^ T-cells, but is dispensable in the absence of inflammation. The ability of IL-2 and IL-15 to induce NKG2D expression on a subset of the resting CD4^+^ T-cells furthermore propose an involvement of the HCMV-primed CD4^+^ T-cells in bystander T-cell activation [45].

It is likely that the NKG2D^+^ CD4^+^ T-cells existing in some pathological diseases are HCMV specific. Interestingly, our analysis of the gene expression profile of NKG2D^+^ CD4^+^ T-cells revealed an increased expression of the two receptors, CX3CR1 and CKMLR1 (Table S1), both of which have been suggested to be involved in the pathology of rheumatoid arthritis [35], [46]–[48].

## Materials and Methods

### Cells

Buffy coats from healthy blood donors were obtained from the Blood Bank at the Copenhagen University Hospital (Denmark) or the Basel Swiss Blood Center. PBMCs were isolated by Ficoll seperation. CD4^+^ T-cells and CD14^+^ monocytes were positively isolated from PBMCs using anti-CD4 and anti-CD14 Ab-coated microbeads (Miltenyi Biotec) respectively, according to the manufacturer’s instruction. The purity of CD4^+^ and CD14^+^ cell isolation was above 98%. PBMCs, CD4^+^ T-cells, and CD14^+^ monocytes were cultured in RPMI 1640 supplemented with 5% human serum, 2 mM L-glutamine, 2 mM streptomycin and penicillin, 1% pyruvate, and 1% nonessential amino acids. A total of nine HCMV seropositive and six HCMV seronegative donors were used in this study. Analysis of serum samples from the blood donors for antibodies against HCMV was done by standard clinical diagnostic tests at Statens Serum Institut (Denmark).

### Reagents and Antibodies

Recombinant IL-2 (proleukin) was obtained from Novartis. Recombinant human TNF-α and IL-15 were purchased from Peprotech. CFSE and dilinoleyloxycarbocyanine (DIO) were purchased from Invitrogen. PI, CHX, sucrose, methyl-β-cyclodextrin, ionomycin, PMA and BrefA were purchased from Sigma. The following antibodies were used for cell sorting and flow cytometry analysis: Rat anti-CCR7 (3D12, IgG2a), anti-rat IgG2a-biotinylated, and streptavidin-pacific blue were kindly provided by Dr. Federica Sallusto (Institute for Research in Biomedicine, Bellinzona, Switzerland). Anti-CD45RA-FITC (ALB11), anti-CD8-Pc5 (B9.11), anti-CD19-Pc5 (J3-119), anti-CD25-Pc5 (B1.49.9), anti-CD56-Pc5 (N901), and anti-CD4-PE (13B8.2) were purchased from Immunotech. Anti-NKG2D-APC (FAB139A), anti-NKG2D-PE (FAB139P), anti-CD71-APC (FAB2474A), anti-NKG2A-APC (FAB1059A), and anti-NKG2C-APC (FAB138A) were purchased from RnD Systems. Anti-CD4-FITC (555346), anti-CD94-PE (555889), and control mouse IgG Abs were purchased from Becton Dickinson. The following antibodies were used for stimulation of cells for cytokine measurements and/or for the redirected cytotoxicity assay: control Ab (mouse IgG) was purchased from Santa Cruz; anti-CD3 Ab (OKT3) was purchased from Imgenex; anti-CD94 Ab (DX22) and anti-NKG2D Ab (5C6) were purchased from eBioscience.

### Cell Sorting

For stimulation with pathogens: Naïve and memory CD4^+^ T-cells were sorted from bead-isolated CD4^+^ T-cells according to the expression of CD45RA and CCR7 surface markers, as previously described [26]. The memory CD4^+^ T-cell pool included both central memory and effector memory CD4^+^ T-cells [26]. For microarray analysis: CFSE-labelled memory CD4^+^ T cells were stimulated with HCMV in the presence of autologous monocytes (as described below) for 20 or 17 days (donor 1 and 2, respectively) before cell sorting. NKG2D^+^ CD4^+^ T-cells were sorted as NKG2D^+^CD4^+^CFSE^–^CD56/8/19/14^–^ and NKG2D^–^ CD4^+^ T-cells as NKG2D^–^CD4^+^CFSE^–^CD56/8/19/14^–^. For cytokine measurements: PBMCs were stimulated with HCMV for 13 days, before sorting of NKG2D^+^ CD94^–^, NKG2D^+^ CD94^+^ or NKG2D^–^ CD94^–^ CD4^+^ T-cells. All samples were stained with 1∶20 diluted Abs in PBS containing 5% human serum and 2 mM EDTA and sorted on a FACSAria (Becton Dickinson) with a purity of >95%.

### In vitro Priming of CD4^+^ T-cells with Inactivated Pathogens

The following inactivated pathogens were kindly provided by Dr. Federica Sallusto (Institute for Research in Biomedicine, Bellinzona, Switzerland) and were used in the following concentrations: *E. Coli* (gram-negative bacterium, 5 particles/monocyte); *S. Aureus* (gram-positive bacterium, 5 particles/monocyte); *S. Pyogenes* (gram-positive bacterium, 5 particles/monocytes); *P. Acnes* (gram-positive bacterium, 2 µg/ml); *M. Tuberculosis* (bacterium, 5 µg/ml); *C. Albicans* (fungi, 5 particles/monocytes); *A. Fumingatus* (fungi, 10 particles/monocyte); *S. Mansomi* (trematode, 5 µg/ml); HCMV (herpesvirus, 5 µg/ml); HSV I+II (herpesvirus, 2.5 µg/ml); VZV (herpesvirus, 5 µg/ml); Influenza (orthomyxovirus, Influvac 09/10, 5 µg/ml); Rubella virus (togavirus, HPV-77, 5 µg/ml); and Mumps virus (paramyxovirus, Enders strain, 5 µg/ml);.

HCMV “cmv 2” (virus infected cell lysate #EL-01-02, strain AD169, 5 µg/ml) was purchased from Microbix, and HCMV “cmv β” (purified β-propiolactone inactivated virus #10-275-500, strain AD169, 2.5 µg/ml) was purchased from Advanced Biotechnologies.

Antigen-specific CD4^+^ T-cell priming was done as previously described [25], with a few modifications. CFSE-labelled naïve or memory CD4^+^ T-cells were co-cultured for 13 days in 96 well microplates (round-bottom) with autologous or allogeneic monocytes (ratio 2∶1) that were pulsed (i.e. pre-incubated) with the indicated inactivated pathogens for 5–6 hr. PBMC cultures (10^5^ cells/well) were incubated with the indicated amounts of HCMV (cmv 2 or cmv β) in 96 well microplates (round-bottom). 50 U/ml IL-2 was added to the samples at day 6 post stimulation, and was supplied whenever new media was added or every 7^th^ day.

### Flow Cytometry Analysis

Cell surface staining with Abs was done as previously described [49]. Cells were stained with 1∶50 or 1∶100 diluted Abs in PBS containing 1% FBS. Intracellular staining was done using the BD Cytofix/Cytoperm kit, according to the manufacturer’s instruction. Cells were stained with 1∶50 diluted Abs in 1× BD perm/wash buffer. Data acquisition was done on BD FACSCanto II or Accuri C6. Data was analyzed using FCS express or CFlow Plus software. All samples were analysed by gating on viable lymphocytes followed by exclusion of duplets. All results show fluorescence on a log-scale.

### Affymetrix GeneChip Human Genome Array

Total RNA was extracted from sorted NKG2D^–^ CD4^+^ T-cells and NKG2D^+^ CD4^+^ T-cells using TRIzol reagent (Invitrogen). Microarray analysis was performed by the RH Microarray Center (Center for Genomic Medicine, Copenhagen University Hospital, Denmark) using a Human Gene 1.0 ST GeneChip array (Affymetrix, Santa Clara, CA, USA). Raw cel files were preprocessed using the Robust Multichip Average (RMA) algorithm including log2 transformation. Probe sets with expression below a minimum detectable limit were omitted from analysis. This limit was set at 5 log2 units, leaving a total of 18665 analysed probe sets. The limit was set based on expression of negative control genes. The microarray data discussed in this publication have been deposited in NCBI’s Gene Expression Omnibus [50] and are accessible through the GEO Series accession number: GSE33670.

### Dynamics of NKG2D Surface Expression

The dynamics of NKG2D and CD71 cell surface expression on HCMV stimulated CD4^+^ T cells following CHX (50 µg/ml) and BrefA (10 µg/ml) treatment was examined as previously described [39]. NKG2D and CD71 surface expression was assessed by flow cytometry 0, 1, 3 and 5 hr post treatment. % of modulation was calculated as: (% expression of treated cells - % expression of un-treated cells)/% expression of un-treated cells * 100.

HCMV stimulated CD4^+^ T cells were cultured in new complete media without cytokines for 24 hr. An aliquot of the samples were analyzed for cell surface expression of NKG2D by flow cytometry. The rest of the cells were transferred to a new well and either left untreated or treated with 50 ng/ml IL-15 or 100 U/ml IL-2+/−0.5 µg/ml CHX for 24 hr, before measuring cell surface expression of NKG2D by flow cytometry.

### Transient Transfections

HCMV-stimulated CD4^+^ T-cells were transfected with the Nucleofector kit (Lonza), accoding to the manufactorer’s protocol. In brief, 10^6^ cells were resuspended in 100 µl nucleofector T cell solution, mixed with 90 nM siRNA, and pulsed with the nucleofector program T-023. CD94 and NKG2D cell surface and intracellular expression was assessed 24 and 48 hr post transfection. Control siRNA (SR30004) and a siCD94 mixture containing three siRNAs directed against CD94 (SR302586A, SR302586B and SR302586C) was purchased from OriGene Technologies.

### Multiplexed Cytokine Analysis

Sorted NKG2D^+^ CD94^–^, NKG2D^+^ CD94^+^ and NKG2D^–^ CD94^–^ CD4^+^ T-cells were stimulated with ionomycin (1 µg/ml) and PMA (0.2 µM) for 5 hr. IgG (2.5 µg/ml), anti-CD3 (20 ng/ml) and/or anti-NKG2D Ab (2.5 µg/ml) was incubated in PBS in a Maxisorp 96 well immune-plate (NUNC) overnight at 4°C. The wells were washed in media to remove unbound Abs. CD4^+^ T-cells transfected with siCtr or siCD94 were stimulated by the plate-bound Abs for 96 hr. The supernatants from each sample were collected and analyzed for cytokine expression using the Diaclone Diaplex kit for human Th1/Th2 inflammation (Gene Probe), according to the manufacturer’s instruction. Data acquisition was done on Accuri C6. Data analysis was done using the FlowCytomix Pro 2.4 software for Th1/Th2 11plex (eBiosciences), with a few modifications. Il-12p70 was deleted from the FlowCytomix Pro 2.4 software and IL-5 and TNF-β were replaced with IL-17A and IL-12, respectively.

### Redirected Cytotoxicity Assay

A redirected cytotoxicity assay [51] was done using allogeneic monocytes as target cells. The target cells were stained with 0.5 mM DiO for 18 hr, before they were washed, counted and incubated with the indicated Ab combinations for 1 hr. Incubation with mouse IgG was used to determine unspecific killing. All Abs were used in a concentration of 2.5 µg/ml. The target cells were then mixed with the effector cells in duplicates. CD4^+^ T-cells (donor 7) stimulated for 9 days with autologous monocytes pulsed with HCMV were used as effector cells. Effector and target cells were mixed in a ratio of E/T: 0, 1 or 2.5 in 200 ul medium in a 96 well microplate (round-bottom). After 5 hr or 24 hr incubation, the cells were stained with PI and immediately analyzed by flow cytometry. To measure the cytotoxic activity, we gated on cells that showed strong DIO-staining and recorded the percentage of PI-positive cells. The Ab-specific cytotoxic activity was found as the cytotoxic activity of a given sample subtracted with the cytotoxic activity of the corresponding IgG sample.

### Statistical Analysis and GSEA

All group comparisons were performed using a Students *t*-test, except the volcano plots in Figure 2, and the results in Table 1 and Table S1, which were calculated with a paired Student’s *t*-test. P-values are reported as uncorrected and considered significant at *p*<0.05, except for the GSEA as described below. When applicable, data are presented as mean±SD.

GSEA was performed using the *gage* algorithm [52] as implemented in R/Bioconductor 2.13.0 [53]. The gene sets used for the analysis were downloaded from the Gene Ontology project [54]. The *gage* algorithm was executed with default settings, except for *same.dir*, which was set to false in order to include sets with both positive and negative regulators. The analysis setup was a paired comparison of all expressed genes between the two sample pairs (i.e. NKG2D^–^ CD4^+^ T-cells and NKG2D^+^ CD4^+^ T-cells) obtained from the microarray analysis of donor 1 and 2. All gene sets reported were significant after correction for multiple testing at a false discovery rate of 5% calculated using the Benjamini-Hochberg method as implemented in the *gage* algorithm. The negative logarithm of this is depicted in Figure 2A.

In Figure 2B volcano plots of selected gene sets were created by plotting the significance of differential expression using a paired Student’s *t*-test as a function of the logarithm of the fold-change between the two groups.

In Table S1, data are presented as the fold change and significance using a paired Student’s *t*-test for the comparison of NKG2D^+^ CD4^+^ T-cells relative to the NKG2D^–^ CD4^+^ T-cells. Table 1 reports genes encoding immune regulatory receptors and Table S1 reports all genes with an average fold change of 1.5 or more between the NKG2D^+^ CD4^+^ T-cells and NKG2D^–^ CD4^+^ T-cells.

## Supporting Information

Table S1 **Microarray.** Reports all genes with an average fold change of 1.5 or more between the NKG2D+ CD4+ T-cells and NKG2D– CD4+ T-cells. Data includes Affimetrix probe set ID, gene symbol, p-value (paired Student’s *t*-test), fold change, mean±SD (log2) for the gene expression values of the NKG2D+ CD4+ T-cells and NKG2D– CD4+ T-cells. (GEO Series accession number: GSE33670).
(PDF)
