# Supplementary material for: Regulation and Gene Expression Profiling of NKG2D Positive Human Cytomegalovirus-Primed CD4+ T-Cells
Source: PLoS One. 2012 Aug 1;7(8):e41577. doi: 10.1371/journal.pone.0041577 (PMC3409864; doi:10.1371/journal.pone.0041577)
Supplement: Table S1 — Microarray. Reports all genes with an average fold change of 1.5 or more between the NKG2D+ CD4+ T-cells and NKG2D– CD4+ T-cells. Data includes Affimetrix probe set ID, gene symbol, p-value (paired Student’s t-test), fold change, mean±SD (log2) for the gene expression values of the NKG2D+ CD4+ T-cells and NKG2D– CD4+ T-cells. (GEO Series accession number: GSE33670). (PDF) [file pone.0041577.s001.pdf]

Table S1

Transcript ID	Gene symbol	P-value	Fold change	NKG2D$^+$ mean±SD log2	NKG2D$^-$ mean±SD log2
7961151	KLRK1	0.124	20.1	10.6 ± 0.1	6.3 ± 1.1
7953949	KLRD1	0.118	10.2	8.4 ± 0.1	5.1 ± 0.8
8173745	CYSLTR1	0.0231	-7.41	4.5 ± 0.3	7.4 ± 0.4
8104901	IL7R	0.121	-5.95	7.2 ± 0.2	9.8 ± 0.5
8043504	MAL	0.00454	-5.42	7.8 ± 0.9	10.2 ± 0.9
7921873	FCGR3A	0.182	5.28	8.6 ± 1.5	6.2 ± 0.5
8122222	PDE7B	0.17	-5.06	5.8 ± 0.7	8.2 ± 0.2
8095343	STAP1	0.00917	-4.51	4.5 ± 0.2	6.7 ± 0.2
7950671	GAB2	0.195	-4.34	6.7 ± 1	8.8 ± 0
8132347	GPR141	0.0139	3.96	8.7 ± 0	6.7 ± 0.1
8069880	TIAM1	0.238	-3.88	5.8 ± 0.2	7.8 ± 1.3
7953333	CD27	0.192	-3.52	6.4 ± 0.1	8.3 ± 0.9
7921868	FCGR3A	0.202	3.48	7.7 ± 1	5.9 ± 0.2
8047677	CD28	0.114	-3.45	8 ± 0.5	9.8 ± 0
8089568	CD200R1	0.103	-3.4	6.6 ± 0.5	8.3 ± 0.1
7903786	CSF1	0.16	-3.36	8.2 ± 0.8	10 ± 1.4
8099471	FGFBP2	0.0435	3.29	10.2 ± 0.1	8.5 ± 0
7973303	TRA@	0.588	-3.16	7.3 ± 2.1	8.9 ± 1
7996081	GPR56	0.164	3.12	8.9 ± 0.9	7.3 ± 0.3
8080562	IL17RB	0.248	-3.1	8.1 ± 0.9	9.7 ± 0
8112107	PPAP2A	0.342	-3.03	5.7 ± 0.1	7.3 ± 1.2
8107307	CAMK4	0.0925	-2.99	7.3 ± 1.1	8.9 ± 0.8
7902205	IL12RB2	0.155	-2.99	7.7 ± 0.4	9.3 ± 0.2
8139100	TARP	0.072	2.97	9.6 ± 0.1	8 ± 0.4
8044021	IL1RL1	0.137	-2.92	4.7 ± 0.8	6.3 ± 0.3
8112865	SERINC5	0.125	-2.91	7.5 ± 0.9	9.1 ± 0.4
8031293	KIR2DL3	0.168	2.88	10.2 ± 1.5	8.7 ± 2.1
8132349	TXNDC3	0.189	2.82	6.5 ± 1	5 ± 0.3
8018975	LGALS3BP	0.111	-2.77	6.9 ± 0.3	8.3 ± 0.1
7951351	PDGFD	0.0642	2.75	7.3 ± 0.6	5.8 ± 0.9
8044035	IL18R1	0.0102	-2.68	6 ± 0.7	7.4 ± 0.8
8047692	CTLA4	0.351	-2.68	6.8 ± 0.9	8.2 ± 0.3
7950555	LRRC32	0.32	-2.67	6.9 ± 0.1	8.3 ± 1
8146500	LYN	0.119	2.66	8.3 ± 0.4	6.9 ± 0
8030782	SIGLEC9	0.0998	2.63	7.8 ± 0.7	6.4 ± 0.4
7931914	IL2RA	0.237	-2.61	8.9 ± 0.4	10.3 ± 0.4
8120783	MYO6	0.0745	2.58	7.5 ± 0.1	6.1 ± 0.1
7986092	FURIN	0.147	-2.58	8.2 ± 0.5	9.6 ± 0.9
8039829	KIR2DL1	0.0627	2.57	8.7 ± 1.4	7.3 ± 1.6
8075310	LIF	0.289	-2.57	5.4 ± 0.1	6.8 ± 0.8
8064485	SIRPG	0.0949	-2.57	7.1 ± 0.1	8.5 ± 0.2
7921677	CD244	0.139	2.56	7.7 ± 0.5	6.3 ± 0
8113369	SLCO4C1	0.119	2.55	8.7 ± 0.7	7.4 ± 0.3
8086344	CX3CR1	0.103	2.51	9.9 ± 0.5	8.5 ± 0.8
7929511	ENTPD1	0.377	-2.46	6.5 ± 0.8	7.8 ± 0.5

8050427	FAM49A	0.151	2.46	8 ± 1.1	6.7 ± 0.7
8031277	KIR2DL3	0.0937	2.46	8.7 ± 1.3	7.4 ± 1.6
7961059	KLRB1	0.263	-2.41	8.3 ± 0	9.5 ± 0.8
7920875	SCARNA4	0.347	2.36	8 ± 0.4	6.7 ± 1.4
7907160	ATP1B1	0.307	-2.35	6.6 ± 0	7.9 ± 0.9
8079377	CXCR6	0.225	-2.35	6.7 ± 1	7.9 ± 0.4
7978544	EGLN3	0.24	-2.35	6.8 ± 1.2	8.1 ± 0.5
8092765	C3orf59	0.0899	-2.34	6.7 ± 0.5	7.9 ± 0.7
7945321	GLB1L2	0.167	2.33	7.7 ± 0.8	6.5 ± 0.3
8169263	VSIG1	0.221	-2.32	5.1 ± 0.6	6.3 ± 1.2
7994280	IL4R	0.163	-2.31	8.4 ± 0.2	9.6 ± 0.7
8055465	CXCR4	0.177	-2.3	7.8 ± 0.3	9 ± 0.2
7988327	PATL2	0.286	2.27	9.9 ± 0.6	8.7 ± 0.2
8106660	RASGRF2	0.0527	-2.27	5.4 ± 0.4	6.6 ± 0.5
7906475	FCRL6	0.165	2.25	8.5 ± 1.3	7.3 ± 0.9
8161906	GNAQ	0.409	-2.23	8.5 ± 2.1	9.6 ± 0.8
8172631	FOXP3	0.356	-2.23	7.1 ± 0.3	8.3 ± 1.3
8039871	KIR3DS1	0.524	2.21	8.2 ± 0.5	7.1 ± 1.3
8139125	TARP	0.0373	2.21	9.6 ± 0.5	8.5 ± 0.6
8084206	B3GNT5	0.442	-2.21	5 ± 0.1	6.1 ± 1.4
8105331	GZMK	0.214	-2.19	10.6 ± 0.5	11.7 ± 0.1
8081214	GPR15	0.123	-2.18	9.4 ± 0.4	10.5 ± 0.7
8150962	TOX	0.0974	2.17	9.7 ± 0.1	8.5 ± 0.4
7918857	TSPAN2	0.325	2.17	7.5 ± 1.3	6.3 ± 0.4
8039842	KIR2DL3	0.225	2.17	8.6 ± 1	7.5 ± 1.6
8123364	CCR6	0.0128	-2.16	5.7 ± 0.3	6.8 ± 0.3
8036503	RASGRP4	0.204	-2.15	6.2 ± 0	7.3 ± 0.5
7968417	FRY	0.0711	2.15	7.8 ± 0.7	6.7 ± 0.5
7972557	GPR183	0.395	-2.13	5.5 ± 0.2	6.6 ± 0.9
7933413	AGAP10	0.148	-2.12	7.1 ± 0.1	8.2 ± 0.3
8152280	LRP12	0.0972	2.1	6.8 ± 0.3	5.8 ± 0.5
7923034	B3GALT2	0.398	-2.1	6.3 ± 2.1	7.3 ± 1
8135488	LRRN3	0.206	-2.1	8.4 ± 2.3	9.5 ± 1.8
8091954	GOLIM4	0.373	2.09	7.2 ± 0.3	6.1 ± 0.7
8058905	CXCR1	0.126	2.08	7 ± 0.5	6 ± 0.2
8031297	KIR2DL1	0.0296	2.08	8.6 ± 1.4	7.6 ± 1.5
7934215	SPOCK2	0.2	-2.08	8.3 ± 0.7	9.3 ± 0.2
7933290	AGAP10	0.135	-2.07	7.3 ± 0.1	8.4 ± 0.2
8133688	SNORA14A	0.477	2.07	8.8 ± 0.1	7.7 ± 1.5
8096116	AGPAT9	0.0418	-2.07	6.4 ± 1	7.4 ± 0.9
8166079	EGFL6	0.151	-2.06	5.5 ± 0.3	6.5 ± 0.6
7961069	CLECL1	0.0609	-2.06	5.5 ± 0.1	6.5 ± 0
8013272	CCDC144A	0.0632	-2.06	5.2 ± 1	6.3 ± 1.1
8011415	P2RX5	0.0233	-2.06	7 ± 0.2	8 ± 0.1
8151512	PAG1	0.294	-2.05	8.7 ± 0.6	9.8 ± 0.1
8055624	ZEB2	0.132	2.05	8.6 ± 0.5	7.6 ± 0.8
7990757	CTSH	0.456	-2.04	7 ± 0.5	8.1 ± 0.7
8031200	KIR3DX1	0.215	2.03	7.5 ± 0.2	6.5 ± 0.7
7969693	RAP2A	0.00109	2.02	10 ± 0.4	9 ± 0.4
8102232	LEF1	0.238	-2.02	6.4 ± 0.3	7.4 ± 0.8

8005204	CCDC144A	0.0328	-2.02	4.9 ± 1	5.9 ± 1
8078442	CCR4	0.407	-2.01	6.3 ± 0.4	7.3 ± 0.7
8162533	PTCH1	0.00683	2.01	9.3 ± 0.5	8.3 ± 0.5
8024572	GNA15	0.115	-1.98	7.4 ± 0.1	8.4 ± 0.1
8070584	TMPRSS3	0.385	1.98	8.3 ± 1.1	7.3 ± 0.1
8166632	GK	0.233	-1.98	6.7 ± 0.9	7.7 ± 0.3
8118137	LTA	0.32	-1.96	8 ± 0.2	9 ± 1
8155673	PIP5K1B	0.272	-1.96	5.5 ± 0.4	6.5 ± 0.3
8107887	CSF2	0.324	-1.96	7.1 ± 0.6	8.1 ± 1.4
8081386	NFKBIZ	0.0926	-1.96	7.2 ± 0	8.1 ± 0.2
7918449	KCNA2	0.576	1.96	7.4 ± 1.7	6.5 ± 0
8178517	NCR3	0.271	1.95	10.3 ± 0.4	9.3 ± 0.2
8179773	NCR3	0.271	1.95	10.3 ± 0.4	9.3 ± 0.2
8124955	NCR3	0.271	1.95	10.3 ± 0.4	9.3 ± 0.2
8113938	ACSL6	0.0228	-1.95	6.1 ± 0	7 ± 0.1
8179258	LTA	0.307	-1.95	8.1 ± 0.2	9 ± 0.9
8151334	MSC	0.128	1.94	8.6 ± 0.6	7.6 ± 0.9
7934161	PRF1	0.0802	1.92	11.6 ± 0.3	10.6 ± 0.2
8148501	PTP4A3	0.114	-1.92	7.4 ± 0	8.3 ± 0.3
7996064	GPR114	0.0751	1.91	9.2 ± 0.2	8.3 ± 0.1
8066925	PTGIS	0.301	-1.91	5.9 ± 0	6.8 ± 0.6
8174103	GK	0.276	-1.9	7.4 ± 0.8	8.3 ± 0.2
7903753	GSTM2	0.019	-1.9	7.7 ± 0.8	8.6 ± 0.9
7989277	MYO1E	0.316	-1.9	6.1 ± 0.4	7 ± 0.3
8146934	LY96	0.229	-1.9	4.9 ± 0.1	5.8 ± 0.4
8177976	LTA	0.308	-1.9	7.8 ± 0.1	8.7 ± 0.8
8014298	C17orf66	0.413	1.89	8.9 ± 0.2	8 ± 0.8
8031311	KIR3DS1	0.301	1.88	7.7 ± 0.1	6.8 ± 0.8
8114938	JAKMIP2	0.114	1.87	7.6 ± 0.4	6.7 ± 0.6
8113433	EFNA5	0.0495	1.87	7.3 ± 0	6.4 ± 0.1
8089112	FILIP1L	0.186	-1.87	5.4 ± 0.3	6.3 ± 0.6
7996377	CES8	0.397	-1.87	7 ± 0.8	7.9 ± 0.1
8043236	GNLY	0.123	1.86	10.9 ± 1.1	10 ± 0.9
8039896	KIR2DS4	0.3	1.86	8.4 ± 0.8	7.5 ± 1.4
8038852	SIGLEC12	0.419	-1.84	6.2 ± 0.3	7.1 ± 0.7
8170119	FHL1	0.0943	-1.84	5.6 ± 0.2	6.5 ± 0.4
8052956	EXOC6B	0.0479	-1.84	7.6 ± 0.2	8.5 ± 0.2
8119223	FLJ45825	0.296	1.84	7 ± 0.3	6.1 ± 0.9
7941444	CTSW	0.0523	1.83	11 ± 0.2	10.1 ± 0.3
7969544	NDFIP2	0.0899	-1.82	7.1 ± 0.6	7.9 ± 0.7
8139107	TARP	0.352	1.82	7.4 ± 1.1	6.5 ± 0.4
7931081	PLEKHA1	0.5	1.82	7.9 ± 0.7	7 ± 0.5
7978272	LTB4R2	0.506	-1.82	7.6 ± 1.5	8.4 ± 0.2
7921900	SH2D1B	0.34	1.81	6.2 ± 0.2	5.3 ± 0.5
8003824	CTNS	0.288	-1.8	7.1 ± 0.1	8 ± 0.7
7975412	TTC9	0.0252	-1.8	7.2 ± 0.1	8.1 ± 0.1
8124166	MBOAT1	0.148	-1.8	8.4 ± 0.5	9.2 ± 0.3
8081171	MINA	0.0502	-1.8	6.1 ± 0.6	6.9 ± 0.7
8088142	CHDH	0.273	-1.79	7.6 ± 0.4	8.4 ± 0.1
8113512	EPB41L4A	0.0844	1.79	6.2 ± 0.1	5.3 ± 0.3

7922219	SELL	0.194	-1.79	6.2 ± 0.4	7 ± 0
8130211	SYNE1	0.0621	1.78	8.3 ± 0.1	7.5 ± 0.2
8140782	ABCB1	0.000589	1.78	7.2 ± 0.9	6.4 ± 0.9
7952884	B3GAT1	0.236	1.78	8 ± 0.5	7.2 ± 0.9
7919872	FAM63A	0.081	-1.78	7 ± 0.2	7.8 ± 0.1
8044333	LIMS1	0.0564	-1.77	8.7 ± 0.2	9.6 ± 0.3
8054519	LIMS1	0.0564	-1.77	8.7 ± 0.2	9.6 ± 0.3
7919645	SV2A	0.0633	-1.77	6 ± 0.4	6.8 ± 0.5
8081341	FAM172B	0.0762	-1.76	5.4 ± 0.6	6.2 ± 0.4
7933772	ANK3	0.168	-1.76	5.7 ± 0.6	6.5 ± 0.2
7975459	SIPA1L1	0.182	-1.75	8.4 ± 0.1	9.2 ± 0.2
8151101	MYBL1	0.289	1.74	10.1 ± 0.1	9.3 ± 0.4
7953943	GABARAPL1	0.302	1.74	7.8 ± 0.4	7 ± 0.2
7973101	RNASE6	0.0574	-1.73	6 ± 0.1	6.8 ± 0
8129637	VNN2	0.254	1.73	6.8 ± 0.9	6 ± 0.5
8059720	NMUR1	0.239	1.73	9.7 ± 0.3	8.9 ± 0.8
8077899	PPARG	0.457	-1.72	5 ± 0.1	5.8 ± 0.9
7908388	RGS1	0.224	-1.72	7.1 ± 1.1	7.9 ± 0.7
8042391	PLEK	0.0739	1.71	9.1 ± 0.2	8.3 ± 0.3
8147503	LAPTM4B	0.123	-1.71	7.9 ± 0.6	8.7 ± 0.4
7898957	RCAN3	0.405	-1.71	8 ± 0.8	8.8 ± 0
7925929	AKR1C3	0.137	1.71	6.1 ± 0.4	5.3 ± 0.2
8100210	TXK	0.382	-1.7	5 ± 0.4	5.8 ± 0.4
8147766	FZD6	0.136	-1.7	5.4 ± 0.4	6.2 ± 0.6
7951413	CARD17	0.241	-1.7	6 ± 0.2	6.8 ± 0.3
8151447	IL7	0.397	1.7	6.4 ± 0.1	5.6 ± 0.6
8060745	SMOX	0.409	-1.7	6 ± 0.3	6.7 ± 0.5
8020806	RNF125	0.372	-1.69	9.3 ± 0.1	10 ± 0.6
8085716	SATB1	0.357	-1.69	8.7 ± 0.6	9.5 ± 0
8078360	STT3B	0.127	-1.69	10.4 ± 0	11.2 ± 0.2
8093294	CCR2	0.174	-1.69	9.5 ± 0.3	10.2 ± 0
7909745	KCTD3	0.398	-1.69	4.9 ± 0.1	5.6 ± 0.7
7906767	FCGR2B	0.179	1.68	7.8 ± 0.4	7 ± 0.1
7980720	TTC7B	0.131	-1.68	6 ± 0.3	6.7 ± 0.1
8157038	SLC44A1	0.225	1.68	7.4 ± 0.5	6.6 ± 0.1
8152522	ENPP2	0.38	-1.68	4.6 ± 0.2	5.4 ± 0.9
7902957	EPHX4	0.218	1.68	7.5 ± 0.7	6.8 ± 0.4
7914112	FGR	0.177	1.67	7.5 ± 0.3	6.8 ± 0
8041781	EPAS1	0.258	-1.67	8.9 ± 0.3	9.6 ± 0.7
8101992	SLC39A8	0.399	-1.67	9.7 ± 0.4	10.5 ± 0.3
8128123	RRAGD	0.327	-1.67	4.8 ± 0	5.6 ± 0.6
7908161	C1orf21	0.0524	1.67	8.8 ± 0.4	8 ± 0.5
8021528	TNFRSF11A	0.198	-1.67	7.6 ± 0.5	8.4 ± 0.2
8128638	SCML4	0.231	1.66	7.2 ± 0.1	6.5 ± 0.3
8176323	IL3RA	0.0278	-1.66	5.9 ± 0.1	6.6 ± 0.1
8165752	IL3RA	0.0278	-1.66	5.9 ± 0.1	6.6 ± 0.1
7944769	GRAMD1B	0.246	-1.66	8.1 ± 0.4	8.8 ± 0
8114572	HBEGF	0.296	-1.65	7.5 ± 0.6	8.2 ± 0.1
7916669	DOCK7	0.147	-1.65	6.2 ± 0.2	6.9 ± 0.4
8148304	TRIB1	0.0258	-1.65	6.2 ± 0.3	6.9 ± 0.2

8128939	TRAF3IP2	0.232	-1.65	6.5 ± 0.1	7.2 ± 0.3
8053882	DUSP2	0.355	-1.65	7.8 ± 0.3	8.5 ± 0.9
8140463	FGL2	0.516	1.65	6.9 ± 0.8	6.2 ± 0.3
7917649	TGFBR3	0.177	1.64	9.9 ± 0.3	9.1 ± 0
7966089	CMKLR1	0.00215	1.64	6.1 ± 0.2	5.4 ± 0.2
8081488	HHLA2	0.329	-1.64	4.8 ± 0.2	5.5 ± 0.3
7958352	BTBD11	0.00641	-1.64	6 ± 0.5	6.7 ± 0.5
7906622	LY9	0.0526	1.64	8.1 ± 1	7.4 ± 1
8079392	CCR2	0.163	-1.64	9.3 ± 0.3	10 ± 0
7963187	LIMA1	0.337	-1.64	8.1 ± 0.2	8.8 ± 0.4
7975453	SNORD56B	0.0499	-1.64	5.4 ± 0.1	6.1 ± 0.1
8116835	GCNT2	0.0816	-1.64	4.8 ± 0	5.5 ± 0.1
8160670	AQP3	0.0276	-1.64	7.6 ± 0.3	8.3 ± 0.3
8172280	SLC9A7	0.378	-1.64	7.2 ± 0.7	7.9 ± 0
8094911	ATP10D	0.154	1.63	8 ± 0.7	7.3 ± 0.4
7975268	ARG2	0.561	1.63	7.5 ± 0.7	6.8 ± 0.5
7935572	PYROXD2	0.167	1.63	6.8 ± 0.4	6.1 ± 0.1
7958960	TPCN1	0.0457	-1.62	6.6 ± 0.4	7.3 ± 0.4
7976292	RIN3	0.264	1.62	9 ± 0.1	8.3 ± 0.5
8099841	TLR6	0.0686	-1.62	5.7 ± 0.5	6.4 ± 0.4
8058091	SATB2	0.0576	1.62	6.5 ± 0.1	5.8 ± 0.2
8114787	GNPDA1	0.0472	-1.62	6.7 ± 0.1	7.4 ± 0.2
8179276	AIF1	0.293	-1.61	5.1 ± 0.5	5.8 ± 0.1
8177996	AIF1	0.293	-1.61	5.1 ± 0.5	5.8 ± 0.1
8118158	AIF1	0.293	-1.61	5.1 ± 0.5	5.8 ± 0.1
8027323	ZNF257	0.491	-1.61	4.8 ± 1.7	5.5 ± 0.8
7963851	KIAA0748	0.226	-1.61	9.2 ± 0.1	9.9 ± 0.2
8106411	S100Z	0.0174	-1.6	5.3 ± 0.3	6 ± 0.3
8030804	CD33	0.259	-1.6	6.4 ± 0.3	7.1 ± 0.7
8094533	DTHD1	0.545	-1.6	5.9 ± 0.9	6.6 ± 2
8117415	HIST1H3E	0.456	1.6	5.3 ± 1.2	4.7 ± 0.3
7953409	PTMS	0.317	1.6	9.7 ± 0.7	9 ± 0.2
8103769	HPGD	0.662	1.59	8 ± 0.4	7.3 ± 2
7991034	HOMER2	0.375	-1.58	7.1 ± 0.1	7.8 ± 0.7
7975779	FOS	0.373	-1.58	6.9 ± 1	7.6 ± 1.6
7907430	FASLG	0.116	1.58	8.6 ± 0.1	8 ± 0
8092009	LRRC34	0.098	-1.58	4.9 ± 0.7	5.6 ± 0.9
7945831	OSBPL5	0.291	1.58	6.7 ± 0.4	6.1 ± 0.1
8170635	ZNF275	0.091	-1.58	7.6 ± 0.1	8.2 ± 0
8031358	KIR2DS4	0.42	1.57	8.3 ± 0.8	7.7 ± 1.5
7951004	C11orf75	0.402	-1.57	7 ± 0.8	7.7 ± 0.2
7939150	PRRG4	0.404	1.57	6.5 ± 0.9	5.9 ± 0.2
7972946	RASA3	0.375	1.57	9.2 ± 0.1	8.6 ± 0.7
7933149	RASGEF1A	0.11	1.57	7.5 ± 0.2	6.8 ± 0
8068353	MRPS6	0.616	1.56	10.5 ± 1.5	9.8 ± 0.2
8088642	LRIG1	0.207	-1.56	8 ± 0.4	8.6 ± 0.1
7983928	LIPC	0.406	1.56	5.4 ± 1.1	4.8 ± 0.4
8086538	LOC644714	0.215	1.56	7.5 ± 0.4	6.8 ± 0.1
7902400	SNORD45B	0.311	1.56	6.5 ± 0.5	5.9 ± 0
8039692	ZNF814	0.42	-1.56	5.9 ± 1	6.6 ± 0.3

7986068	BLM	0.617	-1.55	8.1 ± 1.3	8.7 ± 0
7937782	TSPAN32	0.0475	1.55	8.7 ± 0.2	8.1 ± 0.1
7954245	PLEKHA5	0.255	1.55	7.5 ± 0.4	6.9 ± 0
7996772	SLC7A6	0.374	-1.55	8.6 ± 0.7	9.3 ± 0.1
8120061	ENPP4	0.291	1.55	7 ± 0.9	6.4 ± 0.4
7991224	HAPLN3	0.0206	-1.55	6.2 ± 0.2	6.8 ± 0.1
8110916	LOC442132	0.132	1.54	7.6 ± 0.4	7 ± 0.2
8091778	SCARNA7	0.533	1.54	8.6 ± 0.1	8 ± 1.1
7988444	MYEF2	0.66	-1.54	6 ± 1.7	6.6 ± 0.2
7977270	LOC388022	0.0923	-1.54	8.4 ± 0.1	9 ± 0.2
7962689	VDR	0.219	-1.54	7.3 ± 0	7.9 ± 0.3
8049317	DGKD	0.0538	1.54	8.6 ± 0.4	8 ± 0.3
7944667	SORL1	0.0196	-1.54	9.3 ± 0.2	9.9 ± 0.2
8056217	MXRA7	0.209	1.54	8.1 ± 0.1	7.4 ± 0.2
8141035	SGCE	0.0225	1.54	5.7 ± 0.3	5.1 ± 0.3
7926983	CREM	0.0428	-1.54	6 ± 0.2	6.7 ± 0.2
8059674	GPR55	0.678	1.54	8.2 ± 1.2	7.6 ± 0.4
8088813	PROK2	0.198	1.54	9 ± 0.6	8.4 ± 0.4
7963786	ITGA5	0.485	1.54	9.6 ± 0.9	9 ± 0
8051583	CYP1B1	0.368	-1.54	4.9 ± 0.4	5.5 ± 1
8003158	KIAA1609	0.247	-1.53	6.3 ± 0.1	7 ± 0.5
8109086	ADRB2	0.262	1.53	8.7 ± 0.2	8.1 ± 0.2
8096602	DAPP1	0.238	-1.53	5.9 ± 0.4	6.5 ± 0
7918157	VAV3	0.194	1.53	6.9 ± 0.6	6.2 ± 0.4
8102950	INPP4B	0.00824	-1.53	9.9 ± 0.4	10.5 ± 0.4
8068361	SLC5A3	0.624	1.53	10.8 ± 1.5	10.2 ± 0.2
7946559	GNG10	0.217	-1.53	9.2 ± 0.1	9.8 ± 0.2
8023415	TCF4	0.672	1.52	6.5 ± 1.4	5.9 ± 0.1
7974270	ATL1	0.428	1.52	5.8 ± 0.7	5.1 ± 0
8032682	MATK	0.18	1.52	8.1 ± 0.2	7.5 ± 0
8116859	TMEM14C	0.0135	-1.52	8.1 ± 0	8.7 ± 0
7918379	GSTM3	0.353	-1.52	5.1 ± 0.3	5.8 ± 0.8
7969050	CYSLTR2	0.364	-1.52	4.7 ± 0.2	5.3 ± 0.3
8163629	TNFSF8	0.578	-1.52	9.4 ± 1.4	10 ± 0.3
8114900	PPP2R2B	0.148	1.52	7.6 ± 0	7 ± 0.2
8021301	RAB27B	0.288	-1.52	6.4 ± 0.1	7 ± 0.4
7966052	CRY1	0.48	1.51	7.5 ± 0.9	6.9 ± 0.1
7966441	C12orf47	0.0171	-1.51	6.6 ± 0.5	7.2 ± 0.5
8132013	CHN2	0.0372	1.51	9 ± 0	8.4 ± 0.1
8090591	PLXND1	0.4	1.51	7.7 ± 0.2	7.1 ± 0.4
8048995	ITM2C	0.322	-1.51	8.5 ± 0.2	9.1 ± 0.3
8058477	KLF7	0.141	1.51	9 ± 0.6	8.4 ± 0.4
8042503	MXD1	0.544	1.51	8.8 ± 1.2	8.2 ± 0.2
7919243	CD160	0.475	1.51	5.3 ± 1.3	4.7 ± 0.6
8068713	MX1	0.141	-1.51	6.8 ± 1.2	7.4 ± 1
8136983	OR2A9P	0.068	1.51	7.5 ± 0.2	6.9 ± 0.2
7933427	AGAP4	0.226	-1.51	9 ± 0.1	9.6 ± 0.2
7995096	ITGAM	0.107	1.5	9.9 ± 0.1	9.3 ± 0.2
8022531	NPC1	0.488	1.5	10.1 ± 0.7	9.5 ± 0.2
8151871	CCNE2	0.548	-1.5	7.4 ± 1	8 ± 0.1

8160786	KIAA1161	0.0691	1.5	7.5 ± 0.1	6.9 ± 0
8005687	FAM106A	0.0563	-1.5	6.8 ± 0.9	7.3 ± 0.8
8021442	ZNF532	0.0857	1.5	6.6 ± 0.5	6.1 ± 0.6
7905329	MLLT11	0.43	1.5	8.3 ± 0.3	7.7 ± 0.3
7928671	C10orf57	0.333	-1.5	8 ± 0.9	8.6 ± 0.4
